# Atlanta Academy of Medicine

**Published:** 1876-04

**Authors:** R. W. Westmoreland

**Affiliations:** Reporter


### Atlanta Academy of Medicine.

R. W. WESTMORELAND, M.D., REPORTER.

ATLANTA, February 7, 1876.

Meeting called to order, the President, Dr. H. V. M. Miller, in the chair.

Dr. W. F. Westmoreland presented the case of a negro boy, æt. eight or ten years, affected with contraction of the muscles of one side of the neck. From the history he supposed it was the result of whooping cough. Desired opinions and suggestions of members. Partial paralysis of the vocal muscles exists, preventing perfect speech. The strange feature of the case is that sleep relieves all these symptoms, and even for some short time after awakening the patient is almost en-entirely free from them. Nitrate of silver was given to correct the spasmodic condition, which, as was supposed, resulted from whooping cough.

Dr. Baird was in doubt as to whether the case was an illustration of contraction or contracture of muscles. Would suggest the use of the galvanic current, combined with bromide of potassium.

Dr. Miller, in reference to remarks Dr. Westmoreland had made in regard to giving nitrate of silver when he first saw the patient, inquired to know his object in giving this remedy.

Dr. W. F. Westmoreland—With the idea that the spasmodic condition of the pertussis still existed; the remedy was given as the common treatment in such conditions.

Dr. Miller was inclined to think that paralysis of one side, destroying antagonistic force, might be the cause of the affection, and not spasmodic contraction.

Dr. Armstrong, being called on for his opinion, remarked, that as he did not understand the case, was not prepared to express an opinion.

Dr. Miller inquired if Dr. Westmoreland regarded the disease as local, or resulting from some effusion.

Dr. W. F. Westmoreland regarded it as nervous—a low condition, the result of the cough, which condition he believes is not well understood by pathologists.

Dr. Miller thought that if the idea of the condition being the result of whooping cough was correct, the remedies that were indicated in that disease should be used here.

Dr. Taliaferro thought that as the obscurity of the pathology would prevent a thorough understanding of the case, we should treat the symptoms, and agreed with Dr. Baird in his suggestion.

Dr. Miller was inclined to think that there existed an effusion of the brain, independent of the sequelæ of the whooping cough.

Discussion being exhausted on the above question, Dr. Armstrong called attention of Academy to a remedy that used to be popular in whooping cough, and which he used lately with considerable benefit. This is chestnut leaves in infusion, or decoction. Cited cases in which the disease was very severe, and which recovered in a very short time under the use of remedy. Has had occasion to notice and compare cases where chestnut leaves were used and where they were not used; the latter still whoop, while the former got well readily.

Dr. Taliaferro reported that his experience was not as favorable as Dr. Armstrong's. In some cases the result would be good, and then in others it was without any benefit.

Dr. Armstrong remarked: Several years ago Dr. Smith, of the United States army, stationed at this point, called the attention of this body to a paragraph in one of the medical journals, recommending a syrup made from the leaves of the chestnut tree (castanea vesca) in whooping cough. Shortly after this I had an opportunity to prescribe it in a few cases, and the results were reported to me as very favorable in breaking up the paroxysms. I did not then have an opportunity to verify these reports by personal observation. Within the last three months a number of cases of pertussis have come under my treatment, and in those in which this remedy has been used the results were strikingly good, both in breaking up the troublesome "whoop," as well as in cutting short the milder cough which attends it. I will mention the results in only three families, in which the cases were closely watched by myself.

In the first there were two children, who had the disease distinctly marked by the "whoop." For one of them the remedy in form of syrup was given, and in two days the "whoop" had ceased. The other child took nothing, and was four weeks longer in making a recovery.

Three children, in the second family, had suffered about one month. The youngest, a girl of five years, became so violently ill from the very frequent and prolonged attacks of coughing, that I was consulted. I found, on examination, in addition to the distressing paroxysms of whooping which came on every few minutes, that there was thrown off from the bronchial tubes more than a pint daily of a muco-purulent expectoration. Besides, she had more or less fever every afternoon. The syrup of the chestnut leaves was administered to her, and in twenty-four hours the whooping had greatly diminished, and in three days more it had entirely ceased. From this date convalescence was established. The other two children, who took nothing, continued to have all the symptoms of the disease for several weeks longer.

In the third family, two children, aged respectively four and two years, took this affection at the same time. After having some fever and catarrhal symptoms for about a week, they both commenced to have the characteristic "whoop" of pertussis. The syrup was given as in the other cases, and in two days it had entirely ceased. A slight cough remained two or three days longer, when convalescence was complete.

One would hardly think this a coincidence. In all the cases to which the remedy was administered the results were prompt and striking. On the other hand, those who took nothing continued to suffer. It would seem, therefore, that in this we have a valuable remedy. I hope others will be induced to try this remedy, which will doubtless throw more light on the subject.

In preparing the syrup, a pint of boiling water was used to a large double-handful of leaves. After straining, sugar was added in sufficient quantity to make a syrup. Of this from one teaspoonful or more is to be given every hour.

On motion, Academy adjourned.

---

Atlanta, February 15, 1876.

Academy met, the President, Dr. Miller, presiding.

Dr. J. G. Westmoreland reported the case of a lady aged fifty-seven, who had for several weeks suffered disagreeable nervous symptoms, such as numbness and tingling sensation in the extremities, and occasional vertigo. She was much alarmed at her symptoms, particularly on account of advice which she had received from a homœopathic practitioner to abstain from rich food, from which she inferred that paralysis was threatened. Believing that no primary trouble existed in the nervous system, I instituted investigation, with the view of ascertaining the seat of any local disturbance elsewhere, that might produce the nervous derangement. It was ascertained that digestion was imperfect, and to this the difficulty was referred, until the means successfully used for the restoration of this function failed to give relief from the unpleasant nervous symptoms. About this time, unpleasant symptoms about the bladder, such as strangury, etc., were reported, which led me to suspect some local trouble connected with the pelvic viscera, and suggested to the patient the probability of uterine trouble. This she could not believe, as menstruation had ceased ten years before, and had been impressed always with the opinion that uterine trouble ceased with the "change of life." Further investigation proved the existence of endometritis, which is yielding readily to treatment, and with it the nervous symptoms also disappearing.

This case is reported with the view of calling attention to the prevailing opinion among the people and some physicians, that existing disease ceases with menstruation—whether it ceases from ovariotomy or the natural course of life. The case just reported, and one aged sixty, which I treated last summer with non-malignant ulceration of the os and neck, afford proof against this opinion.

Dr. Kendrick exhibited, for Dr. W. F. Westmoreland, a patient aged thirteen years, who has had pain in his knees since five years ago; by his mother thought to be rheumatism. The internal condyle of right knee is elongated, the external much shortened, the patella partially dislocated outward; has very little use of that leg, which is very much knock-kneed.

Dr. Calhoun thinks that the knock-knee is produced by a relaxation of the internal ligaments of the knee-joint; that the elongation of the internal condyle is due to the fact that, as a result of this relaxation of the ligaments, and consequent turning in of the knee, there is no pressure to prevent its growth, as there is on the external condyle, which is shortened by receiving all the pressure which the entire knee-joint should receive. Has seen knock-knee produced in shoemakers by their constantly turning their feet out, holding between their knees a piece of iron on which to hammer their leather, which position caused an increased pressure on the external condyle, and consequent absorption of that portion of bone.

Dr. Todd asked what dislocated the patella.

Dr. Calhoun said that it was secondary, and the result of the pathological change in the knee-joint and position of the limb, the patella necessarily assuming a position with the ligamentum patella attached below to the tibia. Thinks the only treatment in these cases that could be used is the knock-knee apparatus, but that this patient is too old to expect much benefit to be derived from it.

Dr. Calhoun reported a case of a negro child about eight years old, brought to him by her mother, who stated that she had gotten "live things" out of her left eye.. This statement, notwithstanding its corroboration by an intelligent white lady, he was disposed to doubt, attributing their conclusions to a careless observation. Finding the eye congested, however, with some photophobia, he examined it carefully, but could discover nothing "alive." After repeated search with a magnifying glass, he succeeded in finding and removing some moving substance from underneath the upper lid, which, after examination, proved to be a maggot, exceedingly minute in size. Making several more trials, he removed four or five others. After a few days the congestion of the eye subsided. He accounted for their presence by the fact that a majority of negroes are very uncleanly, and by the filth in this instance a favorable place for the deposition of the eggs of the fly was offered while the child was asleep, and their development into living larvæ. Such a case he had never seen before, nor had he ever read of such an one. He has several times found them in the ear, brought about in the same way as above, but does not know of a case similar to the above. The only treatment, of course, was their removal with forceps.

Academy adjourned.

---

ATLANTA, February 29, 1876.

Meeting called to order by the President, Dr. Miller.

Dr. Armstrong reported a case of hemorrhoids complicated with prolapsus ani, on which he operated. Presented the preserved specimen of the excised tumor to the inspection of Academy. The subject of the disease was sixty-seven years old. Twenty years ago he had internal piles, which occasionally bled, and would protrude when the bowels moved. This was the case for thirteen years; for the last seven years they have protruded in a ring from the anus. Patient stated that during the latter period they would bleed and become very painful. On examination, a ring of hemorrhoids, complicated with prolapsus of mucous membrane of rectum, was discovered. The tumors, as presented, might be called intra-external—attached just within the integumentary margin. On yesterday (Feb. 28) they were removed—in the form of a ring—by the "ecraseur." Very little hemorrhage resulted—less than a tablespoonful. Dr. Armstrong stated that in internal piles he preferred the ligature, as he considered there was less danger of hemorrhage than in the other modes of operating. In this case, as there was no difficulty in the way of controlling the hemorrhage had it occurred, the ligature was not used. In reply to an inquiry if sutures were used, he said that they were not necessary, and had not been used.

Dr. J. M. Johnson remarked that he knew of a case which, on the fourth day after operation by ecraseur, resulted in hemorrhage coming on to a very alarming extent. Mentions the case to show that extraction of tumors by this instrument was not always bloodless.

Dr. Baird requested suggestion from members as to the management of chronic articular rheumatism. He reported a case in which the symptoms are very severe and obstinate.

Dr. J. M. Johnson inquired as to the character of the disease—whether affecting the muscles or joints.

Dr. Baird stated that the case was one of articular rheumatism.

Dr. Alexander suggested fish-brine as a remedy; looked upon by some physicians as almost a specific in such affections.

Dr. Miller remarked, that from the fact that great uncertainty exists as to the pathology of this disease, the treatment was obscure and the theories various. Recounting the various views, he spoke of the rationale of the action of mur. ammonia. This was given to defibrinate the blood in the inflammatory types of the disease, thereby checking the inflammatory process. In reply to an inquiry in reference to the efficacy of local anodyne applications, he remarked that he did not have much faith in any of them. Chloroform possessed his greatest confidence as a liniment.

Dr. Armstrong reported a case of epilepsy, and requested suggestions for the treatment of the same from members of the Academy. The patient, a boy fourteen years of age, was taken in December last with a swoon. One month after had an epileptic fit. Always heretofore healthy. No cause was discovered to which the condition could be attributed. He gave him quinine, zinc, and iron three times a day, with veratrum. This treatment was continued for a month, when he had another convulsion. On the existence of tape-worm being suspected, turpentine was administered. Has since that time been on bromide potassium.

Dr. Baird suggested that the main reliance be placed on the bromides potassium or sodium; commencing with doses of fifteen to twenty grains. Also oxide of zinc, two grains three times daily; not carrying it to the extent, however, of impoverishing the blood. In conjunction with this, suggested strychnia as a tonic.

Dr. J. M. Johnson remarked that he relied on nux vomica and oxide of zinc. Reported a case of a boy sixteen years of age, where the prescription of one-quarter grain of nux vomica and three-quarters grain oxide of zinc were used with good results. No return of fits up to last accounts.

Dr. Baird spoke of the difference of pathology in those cases where the convulsions occurred at night and those occurring in the day, and inquired of Dr. Johnson the condition of his case in this particular. Remarked that strychnia was considered preferable in the nocturnal convulsions, as beneficial in correcting the anæmia of the brain, which is supposed to characterise the nocturnal form.

Dr. Miller inquired if any member had known epilepsy to to be permanently cured.

Dr. Baird was of the opinion that it was sometimes cured.

Dr. Armstrong believed he had cured with quinine and zinc—at least the fits were stopped. Could not say whether permanently or not.

Dr. J. M. Johnson did not remember to have seen but one case cured. Reported a case in which the fits were cured for a year.

Dr. R. W. Westmoreland made the following report of a case of premature rupture of membrane without immediate contraction of the uterus: It is a fact generally conceded by the profession that rupture of the membranes in labor brings on contraction of the uterus. Indeed it is a practice advised by experienced accoucheurs that in an inactive uterus rupture of the membranes should be pursued, as it tends to bring about the activity desired. This result, it would seem, is but natural, since the space occupied by the distended membrane gives occasion to contraction in order to have the partial vacuum occupied. The following case is reported as going to show that not always is the same result to be expected. I was sent for to see a negro woman in labor. When I arrived I found that the os was dilated to the size of a silver quarter of a dollar—pains very active. In making my digital examination, supporting the perineum, and noting the progress of the labor, my attention was incessantly directed to all occurrences, and I therefore, when the labor was completed, felt safe in the conclusion that no water had passed. Upon inquiry the woman told me that two or three days before there had been a gush of water, without pain or contraction of uterus following. I was very particular in my inquiries on this point, and am satisfied that this gush of water was followed by no consequent pain. On the third day after, the pains came on, and there was no difficulty in the labor—dilatation taking place easily, and the contractions of the uterus being rapid and strong. The child was living, healthy, and weighed nine and a half pounds.

Academy adjourned.

